# Excessive Sagittal Slope of the Tibia Component during Kinematic Alignment—Safety and Functionality at a Minimum 2-Year Follow-Up

**DOI:** 10.3390/jpm12091407

**Published:** 2022-08-30

**Authors:** Yaron Bar Ziv, Gilad Livshits, Konstantin Lamykin, Salah Khatib, Yuval Ben Sira, Oded Rabau, Noam Shohat, Ahmad Essa

**Affiliations:** Assaf Harofeh Medical Center, Sackler Medical School, Tel Aviv University, Tel Aviv 6997801, Israel

**Keywords:** posterior tibial slope, arthroplasty, kinematic alignment, reported outcomes, tibia angle

## Abstract

The aim of this study was to assess the safety and functional outcomes of excessive sagittal alignment in the unrestricted kinematic alignment technique for total knee arthroplasty (TKA). A retrospective, single-center study was conducted between 2018 and 2020, including patients undergoing primary TKA with a minimum 2-year follow-up. EOS imaging conducted before and after surgery was reviewed for overall alignment, and a number of measurements were taken, including sagittal tibial slope and other tibia and femur component positioning. Patients were interviewed and asked to fill out several questionnaires including a visual analog scale, the Oxford Knee Score, and the Knee Injury and Osteoarthritis Outcome Score. Overall, 225 patients (66.7%) had a sagittal tibial slope angle above 5° (excessive) and 112 (33.3%) patients had an angle under 5° (moderate). A significant improvement in pain and function scores was observed in both groups following the surgery (*p* < 0.001). There were no significant differences between the moderate and excessive groups in the average VAS, OKS or the various subtypes of the KOOS score. However, there was a slight but significant difference in the number of patients achieving MCID in KOOS symptoms. There were no cases of early failure or loosening. Unrestricted KA and the excessive sagittal alignment of the tibial component seem to be reliable and safe in terms of restoring daily function and alleviating pain after a minimum of 2 years following the surgery.

## 1. Introduction

Total knee arthroplasty (TKA) is considered the gold standard treatment for end-stage knee osteoarthritis [1]. During the surgery, osteoarthritic components are replaced with the aim of achieving a functional and pain-free knee. Although TKA is generally successful, dissatisfaction following TKA is a well-known phenomenon with an estimated rate of 20% following surgery [2,3,4,5,6].

In the last decade, a paramount effort was made to improve the functional outcomes and decrease the dissatisfaction rate following TKA, with the main emphasis on component alignment. Kinematic alignment (KA) was suggested as an alternative technique to traditional mechanical alignment (MA), recreating the pre-arthritic knee’s anatomical alignment with minimal soft tissue manipulation [7], thus promoting more native femur–tibia cycle gait motion.

In recent studies, the employment of KA for TKA has demonstrated promising results in terms of functional outcomes, patient satisfaction, and safety as compared to MA [8,9,10,11]. However, debates in the literature still persist regarding the proper tibial cut technique in the sagittal plane and the need to set a specific cutting limit to avoid excessive posterior tibial slope, with potential subsequent early tibial tray loosening [7,12,13].

In the last 5 years, our institution transitioned from the MA technique to caliper-based nonrestricted KA using the linked technique in which the femur and soft tissue guide the tibial cut [14]; thus, the tibial cut is performed without any restraints. The aim of this study was to evaluate the medium-term safety and patients’ functional outcomes employing this technique and to assess the clinical difference between patients with a tibial cut above and below what is considered excessive in terms of sagittal alignment.

## 2. Materials and Methods

A retrospective, single-center, population-based cohort study was performed between January 2018 and March 2020 to allow for a minimum 2-year follow-up. The extracted data from the hospital electronic registry included all primary TKAs performed by 3 fellowship-trained surgeons during the years the study was conducted. Revision cases, as well as valgus knee arthroplasty cases (*n* = 51), were excluded in this study. Electronic medical records were reviewed for patient age, body mass index (BMI), comorbidities (using the Charlson Comorbidity Index), type of anesthesia (spinal versus general), operative time and length of stay (LOS).

### 2.1. Technique

Starting in January 2018, our institution transitioned from mechanical axis (MA)-based TKA to calipered kinematic alignment (KA) using the linked technique, which we have previously described in detail [14]. In short, the technique involves resurfacing the femur using a conventional calipered technique, which thereafter serves as a guide to cutting the tibia in the coronal plane. Prior to performing the tibial cut, the tibial plateau is thoroughly examined for cartilage remnant. A round and flattened stylus is used as a footprint on an area of cartilage wear in the middle of the medial plateau (where the majority of contact between the femur and tibia occurs) to evaluate the plane of the native slope. A notch is made in the aforementioned orientation and the plane is recreated during the tibial cut. All surgeries were performed with a medial pivot knee design from the same manufacturer. Either cruciate-retaining or cruciate-sacrificing polyethylene was used for all surgeries. No stems or constrained implants were used.

### 2.2. Radiographic Analysis

**Figure 1:**
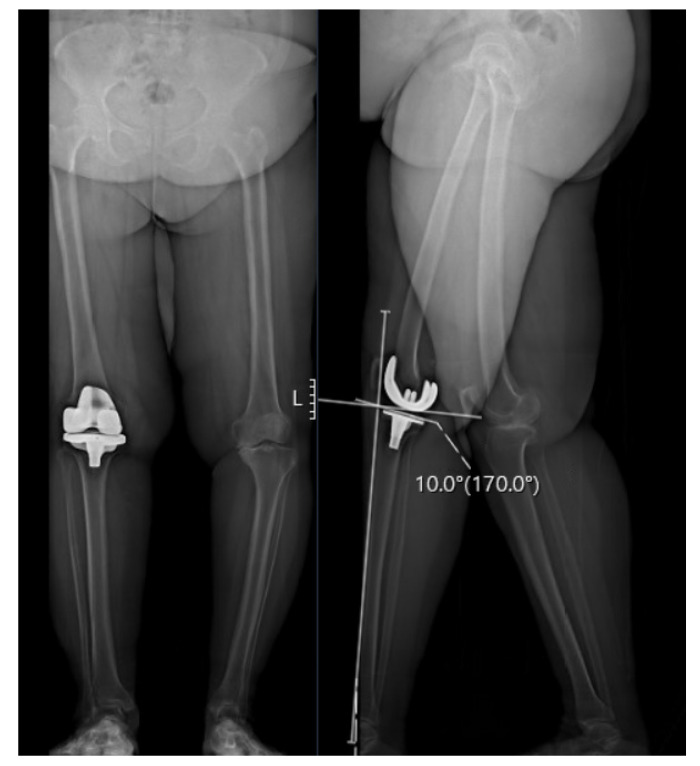
A 73-year-old female with a postoperative PTS of 10°, an MPTA of 81°and an LDFA of 84°. Preoperative VAS was 9, OKS was 20 and overall KOOS was 52 (symptoms 13, pain 32, function 41 and QOL 28). Postoperative scores improved to a VAS of 2, OKS of 45, and overall KOOS of 81 (symptoms 75, pain 89, function 81 and QOL 75) at 2.4 years following surgery.

The preoperative standard protocol included EOS imaging at preadmission testing (2–3 weeks from surgery) at the first follow-up visit after surgery and a two-week following hospital discharge (Figure 1). A number of measurements were performed, including the posterior tibial slope (PTS), medial proximal tibia angle (MPTA), lateral distal femoral angle (LDFA), hip–knee angle (HKA) and tibia bone resorption (TBR) [15,16]. The PTS was measured on a lateral EOS radiograph as the angle between a line perpendicular to the anterior tibial cortex and a line parallel to the tibial plateau/component [17]. Radiographic analysis was performed by 3 orthopedic residents (AE, SH, GL) who were blinded to the clinical outcome assessment. To confirm interobserver reliability, 20 overlapping cases were examined showing a correlation (kappa) of 0.88 (95% confidence interval, 0.79 to 0.96).

### 2.3. Follow-Up Examination

All patients operated on between January 2018 and March 2020 were invited to the follow-up clinic. Those who were not able to attend were phone-interviewed by 3 medical students. A number of patient-reported outcome scores were gathered, including; the visual analog scale (VAS), Oxford Knee Score (OKS), and the Knee Injury and Osteoarthritis Outcome Score (KOOS). Minimal clinical differences for OKS and KOOS were used based on the prior literature [18,19]. Patients were also asked about readmissions and reoperations associated with the operated joint. Range of motion was documented at the most recent clinic visit.

### 2.4. Statistical Analysis

Tibia slope tray angles on the sagittal plane were grouped into 2 categories: moderate (PTS between 0 and 5 degrees) and excessive (PTS above 5 degrees) based on prior publications indicating that 5 degrees is the upper limit for PTS [20,21,22]. Descriptive statistics were calculated for all background characteristics, univariable analysis was conducted using the Chi-square test for nominal data and interval data were analyzed with a T-test for normally distributed data (determined with the Kolmogorov–Smirnoff test) or the Man–Whitney U test (if not normally distributed). The intra- and inter-class coefficients (kappa) were calculated to evaluate the reliability and reproducibility between and within the readers. All analysis was performed using the SPSS packages (version 28.0.1).

The study protocol was approved by the institutional review board as a retrospective medical file study.

## 3. Results

The database search retrieved 385 patients. After excluding patients who did not meet the inclusion criteria, the study’s final cohort included 337 patients. Of these 337 patients, 112 (33.2%) had PTS between 0 and 5 degrees (moderate) and 225 patients (66.8%) had a PTS above 5 degrees (excessive). The time to follow-up was 3.47 years (SD 0.74) for the moderate group and 3.26 years (SD 0.8) for the excessive group (*p* = 0.02). Preoperative pain and functional questionnaires, including VAS, OKS and KOOS scores, were similar in both groups (*p* > 0.05). Additional demographic characteristics, comorbidities and ranges of motion are described in detail in Table 1.

Patients in the moderate group had a smaller mean PTS (9.6, SD 5.4) prior to surgery compared to patients in the excessive group (11.79, SD 5.4) (*p* = 0.001). The average preoperative MPTA, LDFA and HKA were similar in both groups (*p* > 0.5) (Table 2). After surgery, the mean PTS changed to 2.87 (SD 1.8) in the moderate group and to 9.19 (SD 2.9) in the excessive group. (Table 2) In addition, mean LDFA and HKA were significantly higher in the moderate group compared to the excessive group (moderate: LDFA 85.64 SD 4.7 and HKA 2.91 SD 2.9, excessive: LDFA 84.22 SD 3.7 and HKA 1.92 SD 3.44, *p* < 0.05). (Table 2)

**Figure 2:**
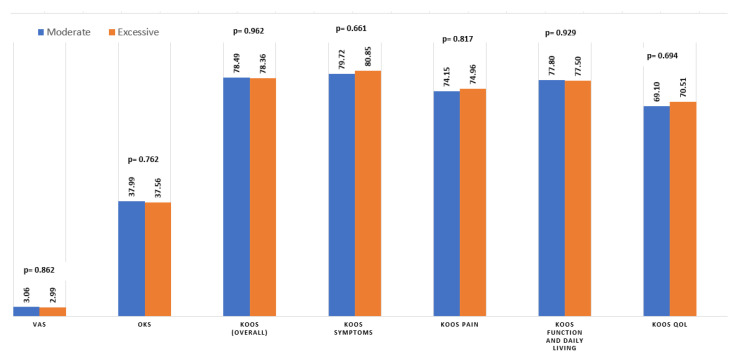
Average patient-reported outcome scores in the moderate versus excessive PTS groups, postoperatively. Abbreviations: (KOOS) Knee Injury and Osteoarthritis Outcome Score; (OKS) Oxford Knee Score; (VAS) visual analog scale; (QOL) quality of life.

A significant improvement in pain and function scores was observed in both groups following the surgery (*p* < 0.001). There were no significant differences between the moderate and excessive groups in the average VAS, OKS or various subtypes of the KOOS (Figure 2). However, there was a slight but significant difference in the number of patients achieving MCID in the KOOS symptoms, as demonstrated in Table 3.

There were also no significant differences in ranges of motion between the two groups; mean extension and flexion ranged between 2.41 (SD 3.81) to 112.58 (SD 13.23) in the moderate group compared to 1.94 (SD 4.16) and 116.25 (SD 15.49) in the extensive group (*p* = 0.495 and 0.146 respectively).

During the study period, two patients required reoperation, one from the moderate group and one from the excessive group (0.4%, *p* = 0.555), due to periprosthetic joint infections. There were no cases of aseptic loosening and the TBR was 2.35 mm (SD 2.21) in the moderate group compared to 3.48 mm (SD 3.58) in the excessive group (*p* = 0.082). There were no cases of instability.

## 4. Discussion

The aim of this study was to assess the functional outcomes and the safety of the excessive sagittal alignment of the tibial component during TKA. To the best of our knowledge, this is the largest study to date to include patients with a PTS larger than 5 degrees. In this study, the employing of excessive sagittal positioning of the tibial component with a PTS above 5 degrees in the KA technique proved to be safe, with comparable objective and subjective patient satisfaction outcomes as reflected by the patient-reported outcome scores, with a mean follow-up time of 3.26 years after surgery.

An optimal prosthetic alignment is mandatory to achieve a stable and functional knee. Unlike the coronal plane alignment, the literature remains heterogenous, with no clear consensus regarding the optimal tibia slope restoration [23,24,25]. The native tibial slope varies between individuals with an average of 3–10 degrees; consequently, attempting a uniform restoration in TKA may lead to unfavorable results [26]. Singh et al., evaluating the impact of preoperative and postoperative PTS differences on postoperative knee flexion, found that a difference larger than 2 degrees in PTS restoration may lead to decreased postoperative knee flexion (<100 degrees) [26]. Catani et al., assessing the stability of tibia implants in regards to the increased difference between the preoperative and postoperative tibial slope, found a negative correlation with tibial component subsidence [27]. Adıyeke et al. compared patients with excessive (>10) and moderate (<5) PTS angles in mobile-bearing TKA and found similar long-term safety profiles and patient-reported outcomes [28]. On the contrary, Nedopil et al., evaluating eight cases of unrestricted KA in a matched case–control study, found a significant association between extreme excessive PTS (>10) and implant failure. However, this study was hindered by a very small study sample and limited confounder control [29]. Still, the literature is very limited regarding safety and patient-reported outcomes regarding native tibia slope restoration, specifically in cases requiring excessive sagittal alignment (above 5 degrees).

The calipered unrestricted KA technique employed in this study is performed using the linked technique. Prior to performing the tibial cut, the tibial plateau is thoroughly examined for cartilage remnants [30,31,32]. A round and flattened stylus is used as a footprint on an area of cartilage wear in the middle of the medial plateau to evaluate the plane of the native slope. A notch is made in the aforementioned orientation and the plane is recreated during the tibial cut. This resulted in the largest cohort to date of patients who underwent excessive sagittal alignment, as 225/337 patients (66.8%) had PTS above 5 degrees.

The findings in this study support the medium-term safety of unrestricted KA technique in general and, in particular, the employment of excessive PTS and native tibia slope restoration in TKA. Restricted KA is usually performed by setting a cutting limit within 5 degrees of the mechanical axis, and HKA must always fall within 3° of neutral in the coronal plane. As for the sagittal plane, a common 3–7-degree limit is employed [33,34]. However, there are no clear, evidence-based principles or specific criteria regarding the optimal sagittal alignment or tibia slope cutting limit present in the literature. This restriction aims to prevent the restoration of extreme outliers in anatomies to, presumably, prevent possible imbalance and early implant failure. However, according to several biomechanical studies, the tibial slope is closely related to the range of motion and patellofemoral alignment; hence, its restoration may lead to improved function and patient-reported outcomes [20,35]. This was demonstrated by Kang et al., using a computational model showing that the maximum force on the quadriceps and the patellofemoral contact stress was decreased as a function of an increased PTS, allowing the patients to feel more comfortable in terms of their knee joint range of motion [20,36]. The results of this study support the medium-term safety and functionality of native PTS restoration in the unrestricted KA technique, with no significant difference in ranges of motion between the moderate and excessive groups.

This study’s advantages include its relatively large study sample of patients with excessive PTS and the number of readers. All measurements were conducted in double-blind settings, thus minimizing the possible reader bias frequently encountered in this type of study.

### Study Limitations

The study’s main limitation is related to its retrospective design and possible recall bias. The only technique employed in this study was KA; consequently, these findings cannot support the safety of excessive PTS in other techniques. Although the follow-up time was limited to 2 years at minimum, with a mean of 3.26 years for the excessive group, it still was not enough time to address and properly evaluate all possible long-term outcomes; hence, the results in this study should be considered preliminary, as a longer follow-up time is needed. Another limitation is related to the fact that all surgeries were performed using the medial pivot prosthetic design of a single manufacturer; thus, further research is needed regarding other manufacturers. Finally, all patients in this study underwent nonrestricted KA surgery, so a comparison of functional results with other alignment techniques is not applicable.

In conclusion, unrestricted KA and the excessive sagittal alignment of the tibial component seem to be reliable and safe in terms of restoring daily function and alleviating pain after a minimum of 2 years following surgery.

## Figures and Tables

**Table 1:** Baseline characteristics, operative factors and patient-reported outcomes in the moderate versus excessive groups.

Variable	Moderate (*n* = 112)	Excessive (*n* = 225)	*p*-Value
Age	69.4 (SD 9.2)	70.98 (7.2)	0.095
Gender (female)	74 (66.1%)	147 (65.3%)	0.497
BMI (kg/m)	31.56 (5.3)	31.79 (5.02)	0.707
CCI	0.88 (1.08)	0.88 (1.16)	0.997
Anesthesia (spinal)	83 (74.1%)	161 (71.6%)	0.36
Operative duration (minute)	83.6 (22.3)	83.25 (20.4)	0.886
LOS	4.25 (2.3)	4.16 (2.6)	0.745
Extension	4.52 (5.9)	3.86 (4.7)	0.469
Flexion	111.29 (15.1)	108.08 (16.6)	0.246
VAS	8.18 (1.4)	8.02 (1.4)	0.349
OKS	12.99 (7.2)	13.8 (7.8)	0.356
KOOS TOTAL	29.36 (15.4)	28.11 (14.6)	0.468
Time to follow-up (m)	41.71 (8.9)	39.17 (9.6)	0.02

Abbreviations: (BMI) bone mass index; (CCI) Charlson Comorbidity Index; (LOS) length of stay; (KOOS) Knee Injury and Osteoarthritis Outcome Score; (OKS) Oxford Knee Score; (VAS) visual analog scale; (m) months.

**Table 2:** Preoperative and postoperative alignment in the moderate and excessive groups.

	Preoperative			Postoperative		
	Moderate	Excessive	*p*-Value	Moderate	Excessive	*p*-Value
PTS	9.6 (5.4)	11.79 (5.4)	0.001	2.87 (1.8)	9.19 (2.9)	<0.001
MPTA	85.14 (3.3)	85.2 (3.6)	0.876	86.14 (3.02)	86.64 (3.1)	0.16
LDFA	89.53 (3.8)	89.46 (4.02)	0.889	85.64 (4.7)	84.22 (3.7)	0.007
HKA	9.47 (4.83)	10.24 (5.02)	0.185	2.91 (2.9)	1.92 (3.44)	0.029

Abbreviations: MPTA (medial proximal tibial angle); LDFA (lateral distal femoral angle); HKA (hip knee angle); PTS (posterior tibial slope).

**Table 3:** Number and percentage of patients achieving minimal clinical differences (MCID) of the Oxford Knee Score (OKS) and the Knee Injury and Osteoarthritis Outcome Score (KOOS) subcategories in the restricted versus excessive groups.

MCID	Moderate (*n* = 112)	Excessive (*n* = 225)	*p*-Value
OKS	68 (60.7%)	128 (56.8%)	0.287
KOOS Symptoms	67 (84.0%)	113 (82.0%)	0.028
KOOS Pain	58 (73.1%)	106 (83.6%)	0.333
KOOS Function	65 (85.3%)	118 (83.6%)	0.238
KOOS QOL	64 (88.5%)	128 (90.2%)	0.276

Abbreviations: (KOOS) Knee Injury and Osteoarthritis Outcome Score; (OKS) Oxford Knee Score; (QOL) quality of life.

## Data Availability

The data presented in this study are available on request from the corresponding author. The data are not publicly available due to privacy and ethical reasons.
